# Successful Electronic Consultation Service Initiative in Quebec, Canada With Primary Care Physicians’ and Specialists’ Experiences on Acceptance and Use of Technological Innovation: Cross-Sectional Exploratory Study

**DOI:** 10.2196/52921

**Published:** 2024-05-30

**Authors:** Véronique Nabelsi, Annabelle Lévesque-Chouinard

**Affiliations:** 1 Department of Administrative Sciences Université du Québec en Outaouais Gatineau, QC Canada; 2 GMF-U de la Haute-Ville du Centre intégré universitaire de santé et des services sociaux de la Capitale-Nationale Sainte-Foy, QC Canada

**Keywords:** eConsult, electronic consultation, digital health solutions, primary care providers, specialists, United Theory of Acceptance and Use of Technology, UTAUT, Task-Technology Fit, TTF, technology acceptance

## Abstract

**Background:**

Electronic consultation (eConsult) is an eHealth service that allows primary care providers (PCPs) to electronically consult specialists regarding their patients’ medical issues. Many studies have demonstrated that eConsult services improve timely access to specialist care; prevent unnecessary referrals; improve PCPs’, specialists’, and patients’ satisfaction; and therefore have a large impact on costs. However, no studies have evaluated PCPs’ and specialists’ acceptance of eConsult services in Quebec, Canada, and worldwide.

**Objective:**

This exploratory study aims to identify factors affecting eConsult service acceptance by PCPs and specialists in urban and rural primary care clinics across 3 regions in the province of Quebec, Canada, by integrating the Unified Theory of Acceptance and Use of Technology and Task-Technology Fit (TTF) models and user satisfaction. This research was designed to broaden and assist in scaling up this effective eHealth service innovation across the province.

**Methods:**

A cross-sectional web-based survey was sent to all PCPs (n=263) and specialists (n=62) who used the eConsult Quebec Service between July 2017 and May 2021. We proposed a unified model integrating the Unified Theory of Acceptance and Use of Technology model and TTF model and user satisfaction by endorsing 11 hypotheses. The partial least squares was used to investigate factors influencing the acceptance of the eConsult Quebec Service.

**Results:**

Of the 325 end users, 136 (41.8%) users responded (PCPs: 101/263, 38.4%; specialists: 35/62, 57%). The results of the analysis with partial least squares method indicate that 9 of our 11 hypotheses are supported. The direct relationships uniting the various constructs of the model highlighted the importance of several key constructs and predominant correlations. The results suggest that satisfaction is the key driver behind the use of the eConsult Quebec Service. Performance expectancy (*P*<.001) and effort expectancy (*P*=.03) can have a positive impact on behavioral intention (BI), and BI (*P*<.001) can impact adoption. TTF has an influence on performance expectancy (*P*<.001), adoption (*P*=.02), and satisfaction (*P*<.001). However, the results show that there is no direct effect between social influence (*P*=.38) and BI or between facilitating conditions (*P*=.17) and adoption.

**Conclusions:**

This study provides a better understanding of the factors influencing PCPs’ and specialists’ intention to adopt the eConsult Quebec Service. Furthermore, this study tests a research model and a technology that have never been explored in Quebec until now. On the basis of the results, the service is a good fit to meet the users’ need to improve access to specialized medical advice. Therefore, the results of our study have made a valuable contribution to the implementation of the service by policy makers in order to maximize acceptance, use, adoption, and success across the province of Quebec. Moreover, after 4 successful years, the eConsult Quebec pilot project is now the Conseil Numérique digital consultation service.

## Introduction

### Background

Access to specialized services remains a significant issue in Quebec, Canada. The Quebec Ministry of Health and Social Services’ (MSSS) 2019 to 2023 strategic plan identified the improvement of access to specialized services, specifically consultations with a medical specialist, as one of the major objectives on which to focus over the next few years [1]. Health care systems face constant pressure to control health care costs while improving access and providing patients with safe, high-quality care. Developing a more efficient and cost-effective health care system is essential to providing better services and a better patient care experience. As part of the 2017 to 2027 Quebec Life Sciences Strategy, the province increased its investment in health research and innovation in order to accelerate the adoption of new and innovative practices [2]. In line with the government’s digital transformation strategy, the MSSS has already begun this transformation within the health and social services network by implementing digital services that will facilitate access to health care and enable rapid and efficient management of patients’ health.

### eConsult Services Worldwide

Today, technological advances and the integration of new practices in the health sector offer interesting possibilities to solve the problem of excessive wait times and equitable access to specialists through digital health solutions [3-7]. Digital health solutions can help overcome barriers to health care access and patient care management. This is especially true for patients living in rural or remote areas who often experience inequitable access to services compared with those living in urban areas [8-13].

Traditionally, patients have in-person consultations with their physicians. Web-based is an alternative method that can be used to improve access and better use specialized resources [14]. Electronic consultation (eConsult) provides an effective and efficient alternative method of assessing a clinical situation without having the patient meet with the specialist in person [3,15-18]. eConsult services are delivered through secure web-based applications that facilitate asynchronous communication between primary care providers (PCPs) and specialists, allowing a PCP to submit a clinical question to a specialist and to get an answer within a week [16,19-21]. The specialist responds with advice on the treatment plan or referral recommendations or a request for additional information about the case. The PCP and specialist continue to communicate until the case is resolved.

eConsult services are spreading rapidly around the world [22] and seeing a significant increase in adoption as a result of the COVID-19 crisis [23-30]. A large body of published academic research has focused on assessing the relevance, usefulness, and impact of eConsult services to reduce wait times for specialist care. Most of the studies were limited to services offering access to a single specialty, such as dermatology [31-34], chronic pain [35,36], obstetrics and gynecology [34,37,38], and others [39-45]. Other researchers have studied PCPs’ [46-48] and specialists’ [16,49-51] level of satisfaction with eConsult services [52]. Studies have also been conducted to examine patients’ care experience, specifically, with regard to access, efficiency, effectiveness, and satisfaction [53-56]. Many studies reported on the impact of the eConsult Service after its implementation, emphasizing growth and sustainability [12,49,50,57-61]. Collected data include the number of web-based consultations submitted by a PCP, the specialties that were consulted, specialist response time, and case outcome. A number of recent studies have evaluated the educational value of the eConsult Service for PCPs [62-65]. Other works also focused on the costs of eConsult services [13,66-68]. A few studies explored the facilitators of and barriers to implementation and adoption of the eConsult Service [23,47,69-72], while other studies focused on the strategies used to disseminate, support, and facilitate efforts to scale up a proven innovation, namely, the eConsult Service, which has improved access to specialized services in primary care settings [73-76].

It is a well-established fact in health-related IT literature that certain factors can influence physicians’ perception of the use of technology such as telemedicine [77-79], electronic medical records [80-83], clinical decision support systems [84-86], and mobile health apps [87-91]. Behavioral acceptance model and technology acceptance model (TAM) are often used to explain user behavior [92-95]. However, no study has ever been conducted on the perceptions and experiences of PCPs and specialists regarding the factors influencing the adoption, acceptance, and use of eConsult services. Research studies are needed to address this gap by exploring the factors influencing the acceptance of eConsult services among health care professionals, thereby contributing to a more comprehensive understanding of technology implementation in health care settings.

The aim of this exploratory study was to fill this gap, integrating variables from 2 models, the Unified Theory of Acceptance and Use of Technology (UTAUT) and Task-Technology Fit (TTF), as well as user satisfaction, to explain user behavior regarding the adoption of the eConsult Quebec Service.

### Research Model and Hypotheses

With the development of IT, many theories and models of the acceptance and use of new technology have been developed or used in order to better understand the factors that influence the acceptance and use of technology [96-101]. Several health sector studies are based mainly on the TAM [93,102-109]. Other works have enhanced the TAM model by creating integrative models that combine the TAM with other explanatory models of user behavior [95,98,110-115]. In view of the multiplicity of existing models on the acceptance and use of technology, Venkatesh et al [100] proposed a unified explanatory model of user behavior, which is the basis of the UTAUT, integrating variables drawn from 8 models (including Theory of Reasoned Action and TAM) [116].

The UTAUT model is considered to be one of the most effective [117] models, and it has been tested and empirically validated in several fields and in different contexts such as health care in order to study the determining factors in acceptance and use of a technological innovation. Moreover, the UTAUT model can explain up to 70% and 50% of the variance in intention to use technology and actual use of the technology, respectively, representing a high predictive power [100,101,104].

At the same time, *fit* is an important notion in IT. It is defined by Goodhue and Thompson [118] as “the degree to which a technology assists an individual in performing his or her portfolio of tasks.” TTF is another theoretical model that has been studied to explain how a new technology leads to performance, to evaluate the impact of adoption, and to assess the relationship between task characteristics (TAC) and technology characteristics (TEC). TTF seeks to assess whether the technology’s functionality is well aligned, that is, compatible with the work done by end users. Studies have shown that technology will be more readily accepted and will have a positive impact on individual performance if the technological characteristics match expected tasks [119-126].

As the TTF model given by Goodhue and Thompson [118] assesses the fit between the task and the technology, it appeared highly relevant to combine the TTF and the UTAUT models because they have the potential to further explain PCPs’ and specialists’ acceptance of the eConsult Quebec Service. In addition, certain studies integrated UTAUT and TTF to understand and predict end user behavior and intention regarding the acceptance and adoption of a new health-related technology [127-133].

Some studies focused on explaining user behavior based on other individual variables such as satisfaction. Resistance to the implementation of new IT can have a negative impact on user satisfaction [134,135]. Resistance may be caused by the perceived risk of performance loss and dissatisfaction following the use of the new IT [136]. The user of an IT seeks to improve their performance by improving the way they work, perform their tasks, and achieve their objectives [118,122,137]. Other studies in the field of health have focused on the application of the TTF model based on end user satisfaction [126,138].

The models’ diversity reflects the existence of several factors influencing IT use behavior, which is a complex and multidimensional phenomenon. Consequently, our study integrated and adapted the UTAUT and TTF models and end user satisfaction with the eConsult Quebec Service.

**Figure 1:**
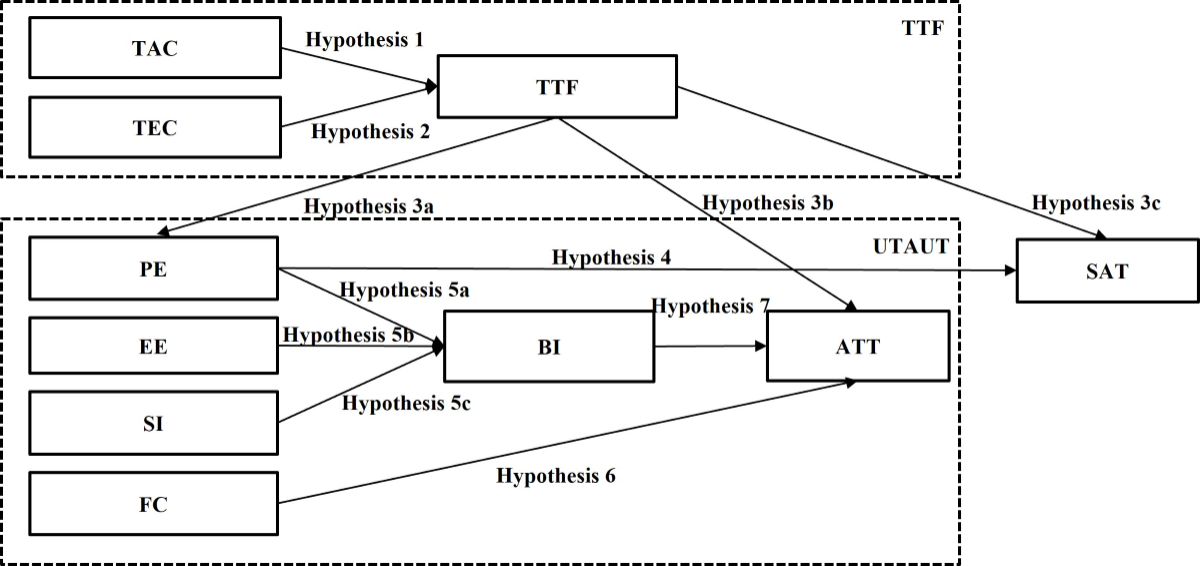
The proposed research model based on Unified Theory of Acceptance and Use of Technology (UTAUT) and task-technology fit (TTF) and user satisfaction. ATT: adoption; BI: behavioral intention; EE: effort expectancy; FC: facilitating condition; PE: performance expectancy; SAT: satisfaction; SI: social influence; TAC: task characteristics; TEC: technology characteristics.

The integrated research framework is presented in Figure 1, and the 11 hypotheses are listed in Textbox 1.

The proposed integrated model includes 10 constructs that have been refined and adapted to the context of the study. The relationships between the variables were hypothesized by referring to previous studies [100,116,118,127-133,138].

**Hypotheses list.**

**Label and hypotheses**

- 1
  Task characteristics have a positive impact on Task-Technology Fit (TTF).
- 2
  Technology characteristics have a positive impact on TTF.
- 3a
  TTF has a positive impact on performance expectancy (PE).
- 3b
  TTF has a positive impact on adoption (ATT) of the eConsult Quebec Service.
- 3c
  TTF has a positive impact on satisfaction (SAT).
- 4
  PE has a positive impact on SAT.
- 5a
  PE has a positive impact on behavioral intention (BI) to use eConsult Quebec Service.
- 5b
  Effort expectancy has a positive impact on BI to use of the eConsult Quebec Service.
- 5c
  Social influence has a positive impact on BI to use of the eConsult Quebec Service.
- 6
  Facilitating conditions has a positive impact on ATT of the eConsult Quebec Service.
- 7
  BI to use eConsult Quebec Service has a positive impact on ATT of the eConsult Quebec Service.

## Methods

### Development of the eConsult Quebec Service

In Ontario, Canada, the Champlain eConsult Building Access to Specialists through eConsultation (BASE) service was initially launched in 2009 to address the issue of long wait times for patients requiring nonurgent care and specialist advice [14,16,139]. The service has demonstrated significant success in reducing wait times, improving access to specialist care, and enhancing patient and provider satisfaction. Given its success in Ontario and in other parts of Canada, the eConsult Quebec Service was based on the Champlain BASE business model and was replicated on an existing telehealth platform within the Quebec health network [3].

Simultaneously, the Quebec team was part of the Canadian Foundation for Healthcare Improvement Connected Medicine collaborative, in partnership with Canada Health Infoway, the College of Family Physicians of Canada, and the Royal College of Physicians and Surgeons of Canada over a period of 18 months from 2017 to 2018 in 7 Canadian provinces (British Columbia, Alberta, Saskatchewan, Manitoba, Quebec, New Brunswick, Newfoundland, and Labrador) to roll out remote consultation services to improve access to specialist medical advice in primary care settings [140].

### Design of the eConsult Quebec Service

The eConsult Quebec Service was adapted to the platform as an additional trajectory among other similar digital consulting trajectories already in operation since 2006. This made it possible to comply with Quebec’s context and security requirements and to offer a simplified user experience, especially for users who were already active on a telehealth platform and did not need to adapt to an additional tool. The new trajectory was integrated into the PCPs’ and specialists’ existing clinical workflow.

Similar to the Champlain BASE eConsult service, eConsult Quebec functions as a platform facilitating communication between PCPs and specialists. This secure web-based application enables PCPs to submit inquiries to specialists, seeking guidance on the best management plan for their patients.

**Figure 2:**
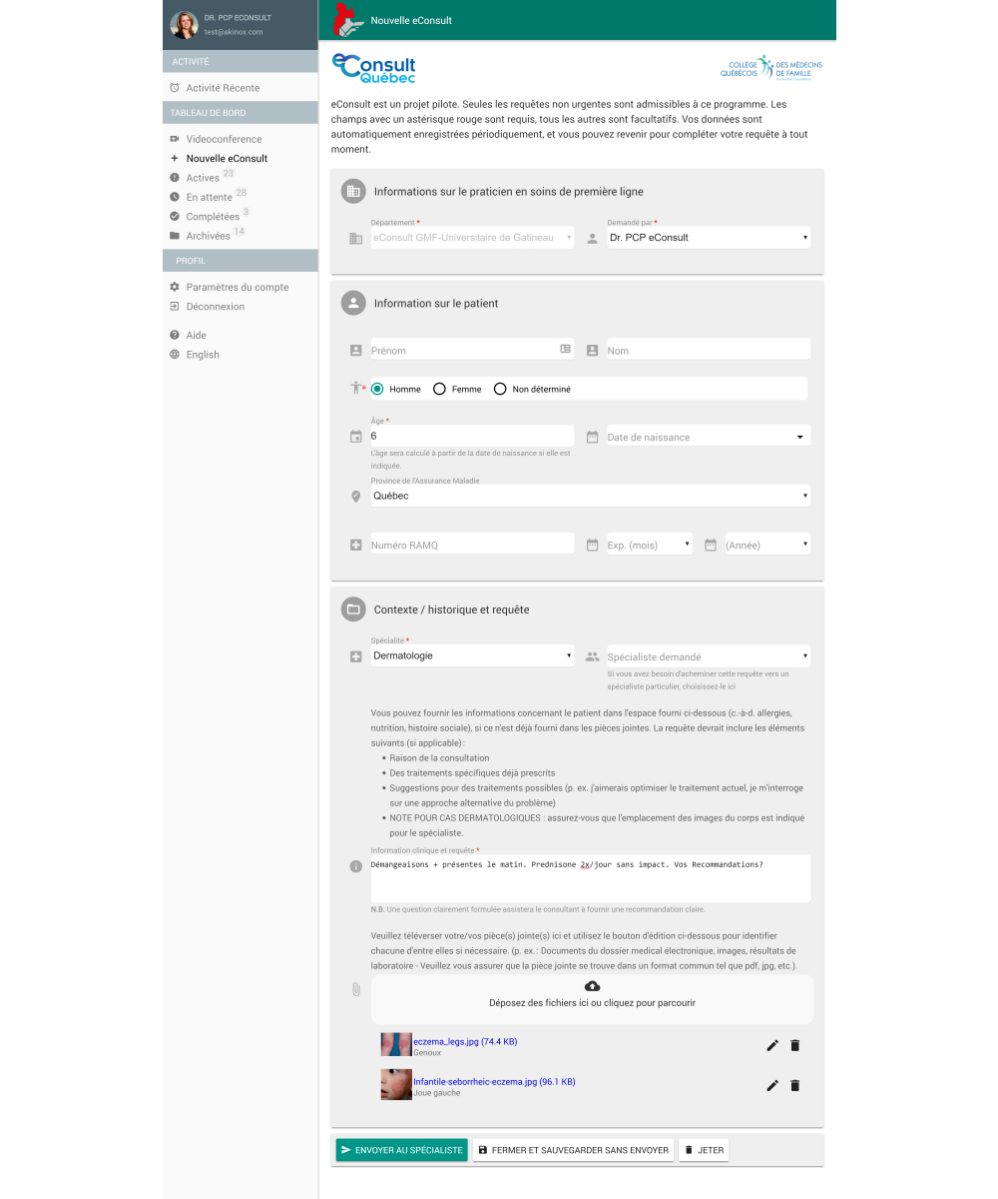
Creation of a new electronic consultation (eConsult) and submission of a clinical question to the specialist.

The platform begins with a PCP submitting a clinical question via a standardized, secure web form (Figure 2). This form is intentionally kept simple and focused to ensure ease of use and favorable user adoption. A centralized coordinator manages all PCPs requests, efficiently dispatching them to specialists of the appropriate specialty. This streamlines the process and ensures timely responses. Upon receiving an email notification, the specialist has a 7-day window to respond to the request and is remunerated at a rate of CAD $200 (US $146.8) per hour, prorated based on their self-reported time needed to address the eConsult.

Furthermore, the platform facilitates clear communication between PCPs and specialists through email prompts and allows for ongoing correspondence until the PCP closes the request. This ensures that all parties involved are kept informed throughout the consultation process. In addition, the patient data are encrypted and accessible only to the physicians involved in the eConsult or their delegated staff through role-based access control. This ensures the confidentiality and security of patient information.

**Figure 3:**
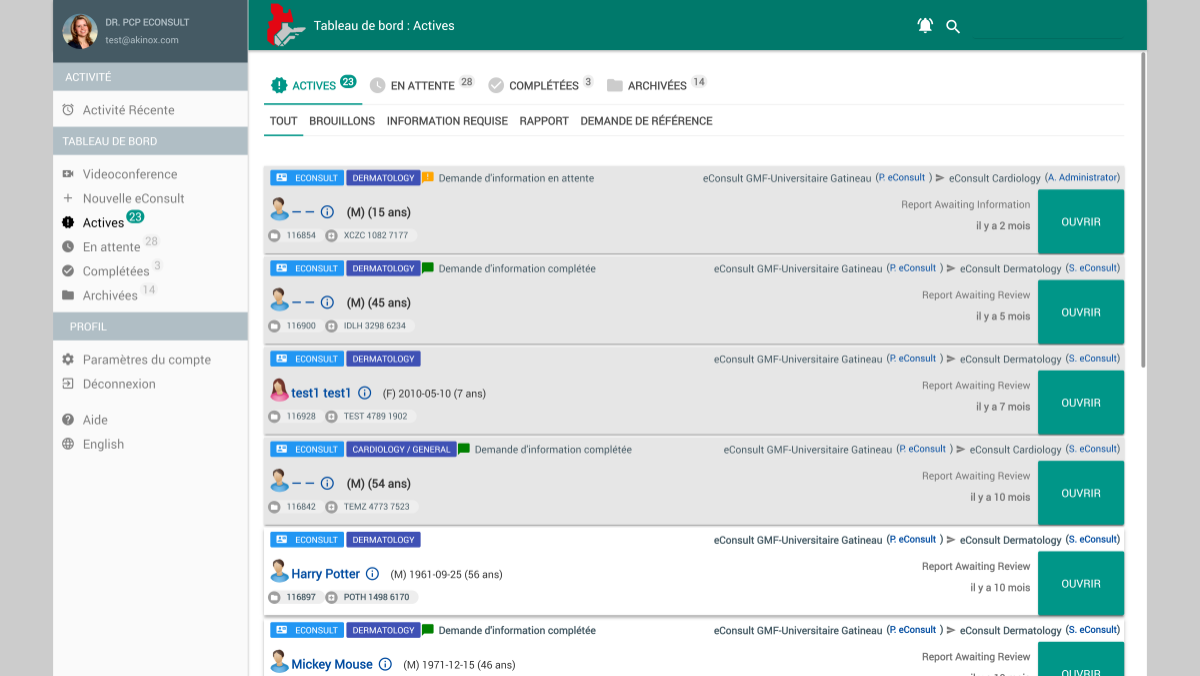
The interface of the primary care providers’ dashboard.

The platform features a user-friendly dashboard interface that consolidates all requests across various communication types, including eConsults, patient forms and messages, teleconsultations, and transfer requests (Figure 3). The view filters and possible actions on the dashboard are dynamically adapted according to user roles and permissions. This ensures that users only see relevant information and functionalities based on their level of access. Users have the option to choose between card view or table view. The main status filters include Active (for pending actions), Waiting (for requests awaiting response), Completed (for finished actions), and Archived (optional storage for completed items).

This adaptation ensured alignment with Quebec’s context and security standards while providing a streamlined user experience, particularly for those already familiar with the telehealth platform. By integrating this new trajectory into the existing clinical workflows of PCPs and specialists, the service facilitated seamless engagement without the need for additional tool adaptation. It is important to note that there are variations in the design of the eConsult service because Quebec is different from other jurisdictions in Canada on a number of fronts, including policy and regulations (eg, licensing, privacy, and liability); financing (eg, provider remuneration); and, of course, language, where French is the national language as opposed to English elsewhere [3].

### Study Design and Setting

Given our objective, we adopted a cross-sectional data collection approach. Kumar [141] explains that cross-sectional design is adequate to study the prevalence, or the occurrence, of a phenomenon, situation, or attitude within a subset of a given population at a certain point in time. In light of this, the study of users’ perceptions, attitudes, and continuance intention with regard to technological innovations lends itself to a cross-sectional study approach.

We conducted a survey-based multicenter cross-sectional study across 3 regions in the province of Quebec (Outaouais, Abitibi-Témiscamingue, and Mauricie-et-du-Centre-du-Québec) as part of the eConsult Quebec pilot project. The web-based survey was sent to all participants (N=325), PCPs (n=263, 80.9%) and specialists (n=62, 19.1%) who used the service between July 7, 2017, and May 17, 2021, in order to assess acceptance and use of the service.

### Instrument

To ensure measurement validity, all items of each model variable were developed based on the UTAUT, TTF, and end user satisfaction. Variables were measured using reliable items that had already been used by previous studies [100,116,118,127-133,138]; we adapted certain items to our study context. The first version of the survey was translated from English into French by a group of researchers and validated by a professional translator to ensure that the content had not lost any of its original meaning. The second and final version of the survey was validated with 4 PCPs and 2 specialists who had already used the eConsult Quebec Service. They were asked to identify any issues that might lead to confusion.

The survey was administered in French to all users of the eConsult Quebec Service. The survey comprised 2 parts. The first part consisted of demographic information (area of origin, profession, and specialty group [for specialists]). The second part included 10 constructs and 38 items, including TAC, TEC, TTF, performance expectancy (PE), effort expectancy (EE), social influence (SI), facilitating conditions (FCs), behavioral intention (BI), adoption, and satisfaction, to measure PCPs’ and specialists’ perception regarding the use of the eConsult Quebec Service. All items were measured on a 5-point Likert scale (1=strongly disagree, 2=somewhat disagree, 3=neutral, 4=somewhat agree, 5=strongly agree). Table 1 presents the items of each construct.

**Table 1:** Items used in the research model.

Construct and item		Measurement
**TAC^a^**		
	JF^b^1	Using the eConsult Service impacts my work performance.
	JF2	Using the eConsult Service can significantly improve the quality of the results of my work.
	JF3	Using the eConsult Service can improve my level of efficiency in the performance of my tasks.
**TEC^c^**		
	FMT^d^1	The way elements are arranged on the eConsult Service screen makes it easy to read the information.
	FMT2	The information in the eConsult Service is clear.
	FMT3	Overall, the information is presented in a useful format.
	SEC^e^1	The risk of an unauthorized third party accessing the eConsult Service is low.
	SEC2	I believe that only the appropriate people have access to information.
	SEC3	I believe that the eConsult Service is secure enough to handle sensitive information.
**TTF^f^**		
	ACC^g^1	The patient information received through the eConsult Service is accurate.
	ACC2	I am satisfied with the accuracy of the information in the eConsult Service.
	ACC3	Overall, I believe the information provided is free of errors.
**PE^h^**		
	PU^i^1	Using the eConsult Service helps me make clinical decisions or offer advice faster.
	PU2	I believe that using the eConsult Service enables me to make safer decisions or offer safer advice.
	PU3	I believe that using the eConsult service enables me to make more accurate clinical decisions or offer more accurate advice.
	PU4	Using the eConsult Service makes my job easier.
	PU5	Overall, I find the eConsult Service to be useful in supporting my clinical decision-making or when offering advice.
**EE^j^**		
	PEOU^k^1	Learning to use the eConsult Service is easy for me.
	PEOU2	My interactions with the eConsult Service are clear and understandable.
	PEOU3	I think that the eConsult Service meets my needs.
	PEOU4	Overall, I find the eConsult Service easy to use.
**SI^l^**		
	SF^m^1	I use the eConsult Service because most of my colleagues use it.
	SF2	My organization facilitated the use of the eConsult Service.
	SF3	My association supports my use of the eConsult Service.
	SF4	The organization generally supported my use of the eConsult Service.
**FC^n^**		
	FC1	I have the knowledge required to use the eConsult Service.
	FC2	My organization supports the use of the eConsult Service.
	FC3	I have the necessary resources to use the eConsult Service.
**BI^o^**		
	BI1	I will continue to use the eConsult Service.
	BI2	I plan to use the eConsult Service frequently.
	BI3	Overall, I think using the eConsult Service is beneficial.
	BI4	I would recommend the eConsult Service to my colleagues.
**ATT^p^**		
	ATT1	Using the eConsult Service is a smart idea.
	ATT2	Using the eConsult Service is a safe experience.
	ATT3	I am in favor of using the eConsult Service.
**SAT^q^**		
	SAT1	Using the eConsult Service meets my needs.
	SAT2	I am happy with my use of the eConsult Service.
	SAT3	I am extremely satisfied with my use of the eConsult Service.

^a^TAC: task characteristics.

^b^JF: job fit.

^c^TEC: technology characteristics.

^d^FMT: format.

^e^SEC: security.

^f^TTF: Task-Technology Fit.

^g^ACC: accuracy.

^h^PE: performance expectancy.

^i^PU: perceived usefulness.

^j^EE: effort expectancy.

^k^PEOU: perceived ease of use.

^l^SI: social influence.

^m^SF: social factors.

^n^FC: facilitating condition.

^o^BI: behavioral intention.

^p^ATT: adoption.

^q^SAT: satisfaction.

### Data Collection

The survey was developed using web-based survey software Survey Monkey (SurveyMonkey Inc). The survey was distributed to all users on May 19, 2021, as an electronic letter through the eConsult Quebec Service electronic mailbox system. The letter included the context, study objective, guarantee of confidentiality, duration, and the link to access the web-based survey. A reminder letter was sent via the electronic mailbox system 1 month after the initial invitation to maximize the response rate.

### Participants

In the end, of the 325 end users, 136 respondents from the 3 regions of Quebec took part in the survey, representing a response rate of 41.8% from both PCPs (101/263, 38.4%) and specialists (35/62, 57%).

### Data Analysis

Several authors argue that the partial least squares (PLS) method is appropriate for exploratory projects and data in various fields [142-146] as well as for the formative measurement of variables that require the operationalization and conceptualization of various concepts [147] in order to “predict and explain a key target construct and to identify its relevant antecedent constructs. In other words, this approach generates latent variable scores that maximize within-sample prediction in terms of the dependent latent variable’s R^2^ value. As such, the estimated coefficients depict the relevance of constructs in a certain model that directly, indirectly and totally contribute to the explanation of a target construct of interest” (Chin et al [148]).

In addition, PLS is appropriate to evaluate relatively new measurement models. Thus, PLS is a promising method for research projects related to information systems and emerging technologies [145], as is the case here. Moreover, this method is especially useful for smaller samples [143] where it may be more difficult to obtain a large number of respondents and where “the goal is to predict and explain the key target constructs or identify the key driver constructs” [149]. This data analysis method is commonly used in research projects based on the UTAUT [150], as well as in studies that combine UTAUT and TTF due to the complexity of the model with several constructs [132,151]. For this reason, this study used PLS to examine the research model and test the 11 hypotheses. Data were analyzed using SmartPLS 4.0 software.

First, as per recommendations from Bagozzi and Phillips [152] and Venkatraman and Grant [153] concerning the preliminary verification of constructs, an evaluation of their validity and reliability was established. Then, the measurement model was evaluated by examining reliability, internal consistency, and convergent validity of measurements. For exploratory research, a Cronbach α coefficient of 0.50 is considered an acceptable value [154-156]. As for the composite reliability index, it must be ≥0.70 [157]. Although Hair et al [155] recommend a factor loading >0.50 to be considered significant, a factor loading >0.40 in the exploratory phase was considered to be a significant contribution [158]. During the reliability test, 2 items (SEC1: “The risk of an unauthorized third party accessing the eConsult Service is low” and SF1: “I use the eConsult Service because the majority of my colleagues use it”) were removed from our research model for data analysis. The validity of constructs was verified using the average variance extracted index, the value of which must be ≥0.50 [159].

Finally, the structural model was evaluated by coefficients of determination (*R*^2^) as well as path coefficients (β) and their significance by running 5000 bootstrap subsamples. Finally, the research hypotheses must be statistically proven with a *t* value of >1.96 and a *P* value of <.05.

### Ethical Considerations

Ethical approval was obtained from the Research Ethics Committee of the Outaouais Integrated Health and Social Services Centre before the beginning of the study (ref. number 2016-183_88), in Quebec, Canada.

All participants gave their consent electronically before beginning the survey. Participation was anonymous and voluntary.

## Results

### Measurement Model Evaluation

As shown in Table 2, for our research model, all factor loading values for all items are >0.40 (0.413-0.931), all Cronbach α values are >0.50 (0.591-0.891), composite reliability values exceed 0.70 (0.783-0.928), and all average variance extracted values are >0.50 (0.550-0.812). These results indicate good reliability and validity of the constructs.

**Table 2:** The measurement model evaluation.

Construct and item			Loadings (>0.40)		Cronbach α (>0.50)		Composite reliability (>0.70)		Average variance extracted (>0.50)
**TAC^a^**					0.659		0.806		0.604
	JF^b^1	0.413							
	JF2	0.913							
	JF3	0.898							
**TEC^c^**					0.790		0.853		0.548
	FMT^d^1	0.822							
	FMT2	0.843							
	FMT3	0.884							
	SEC^e^2	0.563							
	SEC3	0.507							
**TTF^f^**					0.837		0.903		0.758
	ACC^g^1	0.904							
	ACC2	0.913							
	ACC3	0.789							
**PE^h^**					0.891		0.920		0.696
	PU^i^1	0.837							
	PU2	0.838							
	PU3	0.818							
	PU4	0.789							
	PU5	0.886							
**EE^j^**					0.886		0.920		0.741
	PEOU^k^1	0.861							
	PEOU2	0.811							
	PEOU3	0.872							
	PEOU4	0.896							
**SI^l^**					0.742		0.853		0.660
	SF^m^2	0.764							
	SF3	0.799							
	SF4	0.871							
**FC^n^**					0.591		0.783		0.550
	FC1	0.695							
	FC2	0.657							
	FC3	0.856							
**BI^o^**					0.869		0.911		0.720
	BI1	0.850							
	BI2	0.750							
	BI3	0.868							
	BI4	0.917							
**ATT^p^**					0.728		0.844		0.644
	ATT1	0.764							
	ATT2	0.775							
	ATT3	0.866							
**SAT^q^**					0.883		0.928		0.812
	SAT1	0.849							
	SAT2	0.931							
	SAT3	0.920							

^a^TAC: task characteristics.

^b^JF: job fit.

^c^TEC: technology characteristics.

^d^FMT: format.

^e^SEC: security.

^f^TTF: Task-Technology Fit.

^g^ACC: accuracy.

^h^PE: performance expectancy.

^i^PU: perceived usefulness.

^j^EE: effort expectancy.

^k^PEOU: perceived ease of use.

^l^SI: social influence.

^m^SF: social factors.

^n^FC: facilitating condition.

^o^BI: behavioral intention.

^p^ATT: adoption.

^q^SAT: satisfaction.

### Hypothesis Testing

As mentioned, to test our hypotheses, we used the PLS method because it is widely used to test complex causal models, incorporating several latent variables. Sample size constraints are also more flexible, and measurement scales do not require broad approval. Thus, the PLS method is well suited to exploratory analyses. In our structural equation model, path coefficients (β), *t* values, and *P* values are examined in order to distinguish the relationships between the constructs of our research model. In addition, we examined the variance in the dependent variables explained by the independent variables to evaluate the explanatory and predictive power of the structural model (*R*^2^) [160]. According to Falk and Miller [161], the coefficient of determination (*R*^2^) should be >0.10.

**Figure 4:**
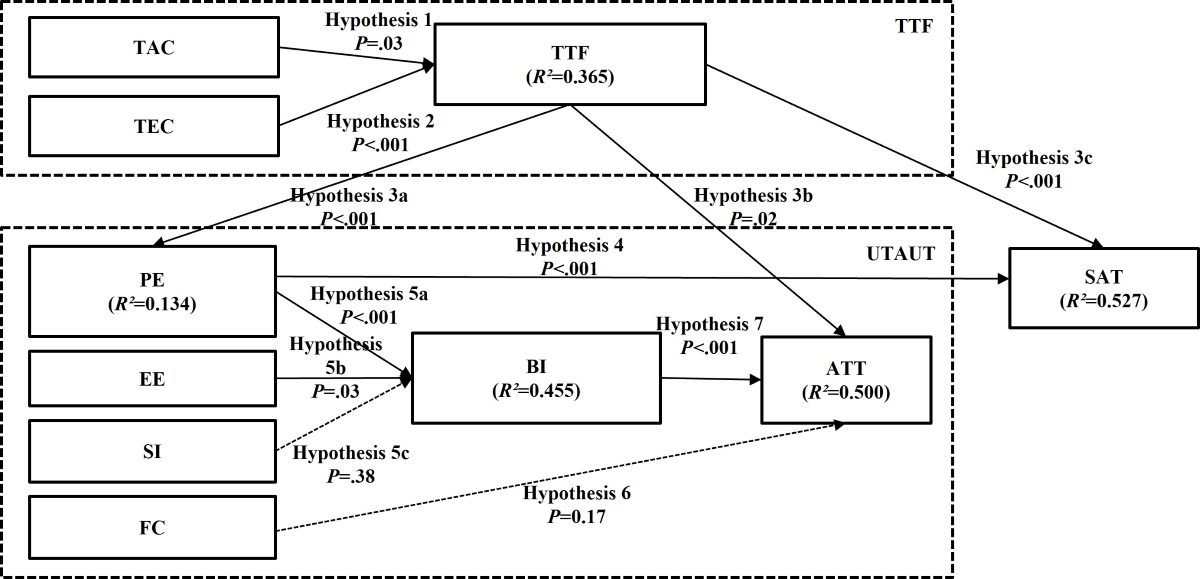
Partial least squares results and R2 values (n=136). ATT: adoption; BI: behavioral intention; EE: effort expectancy; FC: facilitating condition; PE: performance expectancy; SAT: satisfaction; SI: social influence; TAC: task characteristics; TEC: technology characteristics; TTF: task-technology fit; UTAUT: Unified Theory of Acceptance and Use of Technology.

The results of the hypothesis tests on our model integrating the UTAUT, TTF, and user satisfaction with the eConsult Quebec Service are summarized in Table 3 and depicted in Figure 4.

On the basis of Table 3, the statistical result of each path in the research model indicated that most of the hypotheses were supported, except hypothesis 5c effect of SI on BI to use and hypothesis 6 effect of FCs on adoption of the eConsult Quebec Service because the *P* value was >.05 and the *t* value was <1.96.

Both TAC and TEC were found to positively influence TTF, supporting hypothesis 1 (*t* value=2.243; *P*=.03) and hypothesis 2 (*t* value=6.119; *P*<.001). The calculated *R*^2^ values (Figure 2) showed that 36.5% of the variance in TTF was explained by TEC and TAC, with TEC having the strongest influence (*t* value=6.119; *P*<.001). Meanwhile, TTF directly affects adoption, PE, and satisfaction. Thus, hypotheses 3a (*t* value=3.823; *P*<.001), 3b (*t* value=2.415; *P*=.02), and 3c (*t* value=5.604; *P*<.001) were supported. PE has a positive impact on satisfaction, supporting hypothesis 4 (*t* value=4.259; *P*<.001). In addition, TTF and PE explain 52.7% of the variance in satisfaction. Moreover, PE and EE have a direct effect on BI. Thus, hypotheses 5a (*t* value=4.247; *P*<.001) and 5b (*t* value=2.234; *P*=.03) were supported. The 2 variables explain 45.5% of the variance in BI. Finally, BI significantly influences adoption, supporting hypothesis 7 (*t* value=4.861; *P*<.001) and explaining 50% of the variance in adoption. In this model, all the *R*² values of the endogenous variables indicate an acceptable level, respecting the lower limit of 0.10 (TTF, *R*^2^=0.365; PE, *R*^2^=0.134; BI, *R*^2^=0.455; adoption, *R*^2^=0.500; satisfaction, *R*^2^=0.527). Overall, 9 of 11 hypotheses were supported in the research model.

**Table 3:** Hypothesis analysis results.

Hypothesis	Path	β coefficient	*t* value	*P* value	Results
1	TAC^a^→TTF^b^	0.210	2.243	.03	Supported
2	TEC^c^→TTF	0.495	6.119	<.001	Supported
3a	TTF→PE^d^	0.366	3.823	<.001	Supported
3b	TTF→ATT^e^	0.234	2.415	.02	Supported
3c	TTF→SAT^f^	0.498	5.604	<.001	Supported
4	PE→SAT	0.376	4.259	<.001	Supported
5a	PE→BI^g^	0.474	4.247	<.001	Supported
5b	EE^h^→BI	0.257	2.234	.03	Supported
5c	SI^i^→BI	0.064	0.871	.38	Not supported
6	FC^j^→ATT	0.114	1.368	.17	Not supported
7	BI→ATT	0.541	4.861	<.001	Supported

^a^TAC: task characteristics.

^b^TTF: Task-Technology Fit.

^c^TEC: technology characteristics.

^d^PE: performance expectancy.

^e^ATT: adoption.

^f^SAT: satisfaction.

^g^BI: behavioral intention.

^h^EE: effort expectancy.

^i^SI: social influence.

^j^FC: facilitating condition.

## Discussion

### Principal Findings

#### Theoretical Support

To our knowledge, this study is the first to validate both models, namely, UTAUT and TTF, along with user satisfaction to explain user behavior regarding the adoption of the eConsult Quebec Service. The aim of our exploratory study was to identify the factors that predict the acceptance of the service by PCPs and specialists in urban and rural primary care clinics across 3 regions in Quebec, Canada. The results of the PLS analyses indicate that 9 of the study’s 11 hypotheses are supported. To explain the adoption of the service by PCPs and specialists, the direct relationships uniting the various constructs of the model highlighted the importance of several key constructs and predominant correlations, thus confirming most research hypotheses. The research model’s variables influencing each endogenous variable explain 36.5% of the variance in TTF, 13.4% of the variance in PE, 45.5% of the variance in BI, 50% of the variance in adoption, and 52.7% of the variance in satisfaction with regard to the adoption of the eConsult Quebec Service.

First, the results suggest that satisfaction has a solid foundation as a key driver behind the use of the eConsult Quebec Service (*R*^2^=0.527). A few studies in the field of health care technology acceptance have highlighted the pivotal role of satisfaction in predicting individuals’ willingness to adopt technology [95,162,163]. From the perspective of the Information Systems Success Model, individuals’ level of satisfaction can significantly influence the acceptance and use of a particular system [95,117,164-166]. Another study has demonstrated that user satisfaction is the strongest predictor of perceived benefits and technology continuance use intention [163,166,167]. Our model shows that this construct is powered by 2 other constructs that explain 52.7% of the variance: TTF and PE. Studies have shown that PE has a significant impact on satisfaction [168]. According to Bhattacherjee [113], when user expectations are confirmed, they will be satisfied. Thus, PE will affect user satisfaction with a system. Individual productivity is defined as a respondent’s belief in their effectiveness and efficiency and in the quality of their work [122,169]. A user’s perceived feeling of performance in making more accurate and safer clinical decisions or offering more accurate and safer advice (perceived usefulness [PU]; PU2: “I believe that using the eConsult Service enables me to make safer decisions or offer safer advice” and PU3: “I believe that using the eConsult service enables me to make more accurate clinical decisions or offer more accurate advice”) can influence the adoption of the eConsult Quebec Service, how they use it, and the level of satisfaction resulting from their experience with the service. People are happier and more productive when the technology they are using is adapted to their daily tasks. Thus, the better the technology meets the PCPs’ and specialists’ information-related needs when making clinical decisions related to the health of their patients, the more satisfied they are. This correlation shows that the role of TTF affects end user satisfaction. The results suggest that with high levels of TTF and PE, PCPs and specialists experience higher levels of satisfaction.

The results indicate that TAC and TEC explain 36.5% of the variance in TTF for the eConsult Quebec Service. This suggests that both the nature of the tasks being performed and the features and capabilities of the technology platform play crucial roles in determining the overall TTF. Notably, TEC emerged as the factor with the most direct and significant influence on users, as indicated by hypothesis 2 (*P*<.001). This underscores the importance of the eConsult platform’s functionality, user-friendliness, and features in facilitating effective communication and collaboration between PCPs and specialists. In contrast, TAC has a less significant influence, as indicated by hypothesis 1 (*P*=.03). While the nature of the tasks being performed is still important, it suggests that the technology’s capabilities and features are more influential in determining the overall fit and user satisfaction with the eConsult Quebec Service.

With regard to the UTAUT model, PE, EE, and SI explain 45.5% of the variance in BI. BI is the third positive and significant predictor of eConsult Quebec Service use. The most significant direct correlation is between PE (hypothesis 5a; *P*<.001) and the intention to use the technology. The most widely studied relationship in the field of health is that between PE and the intention to use a technology [104,170-178]. Thus, when clinicians’ PE was achieved, it positively influenced their intention to use the system. On the basis of our empirical observations, in the context of voluntary adoption, the correlation between PE and the intention to use the technology is the strongest. The second most frequently measured correlation concerns the impact of EE on the intention to use the system, which has proven to be positively significant in several studies [109,171-174,177,179-182]. In this study, the correlation is weaker but remains positive and significant (hypothesis 5b; *P*=.03). In other words, when considered alone, the user-friendliness of a technology does not totally influence the intention to use a technology, rather the technology must also be perceived as useful. On the basis of our field data, we expected to see a weaker correlation between EE and BI to use the eConsult Quebec Service than between PE and BI to use the service. Regarding the role of SI in relation to intention to use the system, the reviewed studies tested a positive correlation between these 2 constructs [183]. Note that SI has no direct effect on this factor (hypothesis 5c) in our study. As a first line of thought, it is possible that physicians as self-employed workers within the public health system in Quebec are less sensitive to social pressure when using technology. As a second explanation, it is possible that this finding reflects the voluntary context of the adoption and use of the eConsult Quebec Service by PCPs and specialists. As a third explanation, it is possible that health care professionals may rely more on their individual judgments and preferences rather than being influenced by social factors. In certain contexts, the influence of peers or colleagues on decision-making may be limited.

Adoption is the second predictor of the use of the eConsult Quebec Service. Our model demonstrates that this construct is powered by 3 other constructs that explain 50% of the variance: BI, FCs, and TTF. The most significant direct correlation is BI (hypothesis 7; *P*<.001). Some studies have noted that a person’s emerging intention represents a critical point during the adoption of a technology, which may be considered as the moment when the decision is made to accept the change and modify their behavior [104,170,172,184]. Thus, attitude toward technology lies in the intention to use the technology. The second direct but weaker correlation is TTF (hypothesis 3b; *P*=.02). Several studies have confirmed the importance of TTF in the adoption of a new technology [119-121,127,185]. Generally, the compatibility of IT with the preferred work style and current work practices would influence the adoption of the IT [130]. As for the last construct, FCs have no effect on the adoption of a system (H6: β coefficient=0.114). Therefore, this result suggests that PCPs and specialists are comfortable with the technology, have confident in the availability of support measures to assist them in case of issues, and feel adequately informed and competent when adopting the eConsult Quebec Service.

#### User Adoption

This construct is defined as the user’s perception of the factors in the work environment that promote the adoption and use of the service, namely, with regard to the available support, coaching, and training provided. This means that all the barriers hindering the adoption of a technology were eliminated by the team during the roll out of the eConsult Quebec Service. Initially, the pilot project was launched in the Outaouais region with 1 clinic; other clinics were added during the deployment phase in this region. This approach made it possible to control recruitment, and, above all, it facilitated PCPs’ and specialists’ ownership of the use of the service as well as enable the validation of monitoring mechanisms. Subsequently, the project was rolled out in several clinics simultaneously in 2 other regions (Mauricie-et-Centre-du-Québec and Abitibi-Témiscamingue).

The operation of the service is very simple and user-friendly [3]. More precisely, the platform begins with a PCP submitting a clinical question via a standardized, secure web form. This form is intentionally kept simple and focused to ensure ease of use and favorable user adoption. A centralized coordinator manages all PCPs requests, efficiently dispatching them to specialists of the appropriate specialty. This streamlines the process and ensures timely responses. Also, the platform facilitates clear communication between PCPs and specialists through email prompts and allows for ongoing correspondence until the PCP closes the request. This ensures that all parties involved are kept informed throughout the consultation process. In addition, the patient data are encrypted and accessible only to the physicians involved in the eConsult or their delegated staff through role-based access control. This ensures the confidentiality and security of patient information.

The platform features a user-friendly dashboard interface that consolidates all requests across various communication types, including eConsults, patient forms and messages, teleconsultations, and transfer requests. The view filters and possible actions on the dashboard are dynamically adapted according to user roles and permissions. This ensures that users only see relevant information and functionalities based on their level of access.

Given the user-friendliness of the platform and available tools (video, PowerPoint presentation, and test platform), no specific training is required. Users contact the resources responsible for the project in their region as needed. Therefore, the system itself requires little user support. As part of the pilot project, the team made sure that users could operate with maximum autonomy. Thus, PCPs and specialists were comfortable using the eConsult Quebec Service.

#### Integrating Theoretical Models

The relationship between the TTF model and the UTAUT model is demonstrated by the influence of TTF on PE. Indeed, TTF has a positive and significant effect on PE (hypothesis3a; *P*<.001), and TTF explains 13.4% of the variance in PE. This finding is similar to other studies [130,186]. Thus, the authors found that a technology can have a positive impact on performance when it is used and aligned with the supported task [118].

In the context of this study, the higher the TTF level, the more PCPs and specialists perceived the eConsult Quebec Service as useful and conducive to making work easier. Thus, the technology’s fit to user requirements would directly influence their expectations. During the pilot project, integration of the eConsult Quebec Service into health care practices was identified by PCPs and specialists as an important issue likely to affect clinical practices, as well as the safety and the quality of care provided to patients. Studies carried out in health care settings clearly indicate that during its adoption, the compatibility of a technology influences PE [122,137,171,174,178,187-190].

To this end, deployment of the service made it possible to optimize the consultation process following an eConsult and to provide the patient with optimal care management following an eConsult, and in most cases, the PCP’s action plan was improved. Overall, PCPs and specialists found the eConsult Quebec Service to be useful in supporting their clinical decision-making or when offering advice (PU5). Moreover, the correlation between the 2 models is presented by the influence of TTF on adoption. The greater the alignment between the technology and the task at hand, the more likely the user will be to adopt and use the technology. As mentioned, the results indicate that TTF contributes little to explaining the attitude toward actual use of the eConsult Quebec Service (hypothesis 3b; *P*=.02). Note that user perception can be affected by various factors, namely, the voluntary nature of adopting a technology. Also, as part of the pilot project, adopting the eConsult Quebec Service was not mandatory, so PCPs and specialists used it on a voluntary basis.

### Limitations and Future Research

This study has some limitations that can be addressed in future studies. First, the results of this study may be difficult to generalize since the study was conducted in only 3 regions across Quebec. In addition, the Quebec MSSS selected these 3 regions for the pilot project with the aim of improving access to specialized medical advice. In future studies, it would be relevant to extend the scope to a larger population of PCPs and specialists from different regions in Quebec to increase the generalizability of the findings. This would help to confirm the model and its research variables relating to the use of the eConsult Quebec Service on a larger scale.

Second, this study did not compare the user groups, that is, PCPs and specialists, because the objective was to study the applicability of the model integrating UTAUT and TTF and end user satisfaction with the eConsult Quebec Service. However, perception of the service may be different from one group to another.

Third, this study did not delve into the demographic characteristics of the participants. It would be interesting to explore in future research whether the factors motivating the adoption of eConsult Quebec Service differ among different groups of health care professionals.

Fourth, the research design used in this study was cross-sectional, capturing PCPs’ and specialists’ overall experience at a specific point in time. A longitudinal study would enhance the robustness of our findings by tracking experiences over an extended period. This longitudinal study would not only strengthen the validation of our research model but also allow for a deeper understanding of how perceptions and use of eConsult Quebec Service evolve over time.

Fifth, this study did not find support either for the effect of SI on BI to use of the eConsult Quebec Service (hypothesis 5c) or for the effect of FCs on the adoption of the eConsult Quebec Service (hypothesis 6). In future research, it would be relevant to explore these 2 factors through interviews with PCPs and specialists in Quebec.

Sixth, all the reliability and validity analyses demonstrated satisfactory results, with the exception of 2 items: one from the SI construct (social factors: SF1: “I use the eConsult Service because the majority of my colleagues use it”), and the other from the TEC construct (security: SEC1: “The risk of an unauthorized third party accessing the eConsult Service is low”), which were withdrawn due to low factor loading.

Finally, this study did not investigate potential barriers to the adoption of eConsult Quebec Service. Thus, future studies should identify these barriers, as doing so could provide valuable insights for policy makers and health care administrators. By understanding the obstacles hindering adoption, effective strategies can be developed to address them.

### Conclusions

This study has used an extended model that combines the UTAUT and TTF, along with user satisfaction, to delve into the factors influencing the intention of PCPs and specialists in 3 regions across Quebec to adopt the eConsult Quebec Service. In addition, this study has tested a research model and a technology that have not previously been explored in Quebec, Canada. Given that most of our research hypotheses were validated by the study’s outcomes, it can be asserted that our integrated research model is highly relevant to the study’s context. In this regard, the eConsult Quebec Service is well suited to the users’ need to improve access to specialized medical advice.

By integrating well-established theoretical models such as the UTAUT and TTF, our study offers a comprehensive theoretical perspective to understand the factors influencing the acceptance of the eConsult Quebec Service. By grounding our analysis in these frameworks, we were able to identify and explore the factors shaping acceptance behavior among health care providers. This study has demonstrated the significance of certain factors that contribute heavily to actual use of the service. Indeed, our results indicate that TAC, TEC, TTF, PE, EE, BI, adoption, and satisfaction have an effect on users of the eConsult Quebec Service, whereas SI and FCs have no significant impact. Note that satisfaction is a significant predictor, essential to evaluating the acceptance of the eConsult Quebec Service. PE and EE can positively predict BI, and BI can impact adoption. In addition, TTF has an influence on PE, adoption, and satisfaction.

Also, by focusing on PCPs and specialists in both urban and rural settings across different regions of Quebec, our study provides valuable insights into the specific factors influencing acceptance within this diverse health care landscape. This context-specific understanding is important for tailoring interventions to address the unique needs and challenges faced by health care providers in different settings.

The main conclusion of this study emphasizes the significance of identifying the key factors influencing the adoption of the eConsult Quebec Service before scaling up the service. This has implications for improving patient access to specialist care, enhancing care coordination, and optimizing health care resource use. Our study findings directly have provided policy makers with valuable insights before the service’s implementation across Quebec, aiming to enhance its acceptance, use, adoption, and success. In fact, after 4 successful years, the eConsult Quebec pilot project is now the Conseil Numérique digital consultation service. This new service, launched in December 2021, has been rolled out across Quebec by the MSSS. The Conseil Numérique digital consultation service promotes swift communication between PCPs and specialists by providing timely access to specialist medical advice. Thus, it contributes to better case management by the PCP. So far, >30,000 cases have been processed following the scaling-up of the consultation service since December 2021. In >40% of eConsult cases, an in-person visit to the specialist was considered but deemed unnecessary following the eConsult. This reduced unnecessary wait times for patients and freed up specialist resources for patients who needed them most. When a patient needed a visit with a specialist, those visits were often more efficient and productive due to the steps or treatment initiated through the eConsult before the in-person visit.

## Abbreviations

BASE: Building Access to Specialists Through eConsultation
BI: behavioral intention
eConsult: electronic consultation
EE: effort expectancy
FC: facilitating condition
MSSS: Ministry of Health and Social Services
PCP: primary care provider
PE: performance expectancy
PLS: partial least squares
PU: perceived usefulness
SI: social influence
TAC: task characteristics
TAM: technology acceptance model
TEC: technology characteristics
TTF: Task-Technology Fit
UTAUT: Unified Theory of Acceptance and Use of Technology
